# Remission Induced by TNF Inhibitors Plus Methotrexate is Associated With Changes in Peripheral Naïve B Cells in Patients With Rheumatoid Arthritis

**DOI:** 10.3389/fmed.2021.683990

**Published:** 2021-06-17

**Authors:** Borja Hernández-Breijo, Chamaida Plasencia-Rodríguez, Victoria Navarro-Compán, Carlota García-Hoz, Israel Nieto-Gañán, Cristina Sobrino, Javier Bachiller-Corral, Mariana Díaz-Almirón, Ana Martínez-Feito, Teresa Jurado, Paloma Lapuente-Suanzes, Gema Bonilla, Cristina Pijoán-Moratalla, Garbiñe Roy, Mónica Vázquez-Díaz, Alejandro Balsa, Luisa M. Villar, Dora Pascual-Salcedo, Eulalia Rodríguez-Martín

**Affiliations:** ^1^Immuno-Rheumatology Research Group, Hospital La Paz Institute for Health Research-IdiPAZ, Madrid, Spain; ^2^Rheumatology, La Paz University Hospital, Madrid, Spain; ^3^Immunology, Ramón y Cajal Institute for Health Research, Hospital Universitario Ramón y Cajal, Madrid, Spain; ^4^Rheumatology, Ramón y Cajal Institute for Health Research, Hospital Universitario Ramón y Cajal, Madrid, Spain; ^5^Biostatistics Unit, Hospital La Paz Institute for Health Research-IdiPAZ, Madrid, Spain; ^6^Immunology, La Paz University Hospital, Madrid, Spain

**Keywords:** rheumatoid arthritis, autoimmunity, B cells, remission, TNF inhibitors

## Abstract

Biological therapies, such as TNF inhibitors (TNFi), are increasing remission (REM) rates in rheumatoid arthritis (RA) patients, although these are still limited. The aim of our study was to analyze changes in the profile of peripheral blood mononuclear cells (PBMC) in patients with RA treated with TNFi in relation to the clinical response. This is a prospective and observational study including 78 RA patients starting the first TNFi. PBMC were analyzed by flow cytometry both at baseline and at 6 months. Disease activity at the same time points was assessed by DAS28, establishing DAS28 ≤ 2.6 as the criteria for REM. Logistic regression models were employed to analyze the association between the changes in PBMC and REM. After 6 months of TNFi treatment, 37% patients achieved REM by DAS28. Patients who achieved REM showed a reduction in the percentage of naive B cells, but only when patients had received concomitant methotrexate (MTX) (OR: 0.59; 95% CI: 0.39–0.91). However, no association was found for patients who did not receive concomitant MTX (OR: 0.85; 95% CI: 0.63–1.16). In conclusion, PBMC, mainly the B-cell subsets, are modified in RA patients with TNFi who achieve clinical REM. A significant decrease in naive B-cell percentage is associated with achieving REM after 6 months of TNFi treatment in patients who received concomitant therapy with MTX.

## Introduction

Tumor necrosis factor inhibitors (TNFi) are widely used for the treatment of patients with rheumatoid arthritis (RA), who do not respond to conventional synthetic disease-modifying antirheumatic drugs (csDMARDs). However, 20–40% of patients do not achieve an adequate clinical response to TNFi (1). Moreover, despite the continuous advances in the understanding of the molecular mechanisms underlying the effect of TNFi therapy, there is still a lack of objective parameters associated with clinical response to TNFi in RA (2, 3).

It is well-known that different types of immune cells such as monocytes and natural killer (NK), T, and B cells are involved in the development of inflammation and autoimmunity in the RA pathogeny (4–6). Although these cells are recruited to inflamed synovium, peripheral blood mononuclear cells (PBMC) may be reflecting the local inflammation, thus providing useful information about the disease status. Accordingly, several publications have reported that TNFi treatment could modify the proportion of the different PBMC subsets (7–9). The effect of TNFi on B cells has not been extensively studied and controversial results have been reported. On the one hand, published data showed that TNFi treatment may increase the frequency of CD27^+^ memory B cells in responders (10). On the other hand, other studies described reduced CD27^+^ or double negative IgD^−^ CD27^−^ memory B cells as a consequence of TNFi therapy (11, 12). In a different study, the frequency of IL-10 producing regulatory B cells (B10 cells) appears to be increased by TNFi treatment in responders (9). We have recently published that patients who did not achieve remission by Disease Activity Score-28 (DAS28) after 6 months of TNFi therapy showed lower percentages of total and naive B cells at baseline than remission (REM) subjects. However, to deepen into this field, more studies are needed (13).

In this study, we aimed to analyze whether treatment with TNFi modulates PBMC in relation to clinical response in patients with RA.

## Materials and Methods

### Patients

This was a prospective, observational, longitudinal bi-center pilot study, including 78 patients with RA starting a first TNFi [infliximab (*n* = 14), adalimumab (*n* = 3), etanercept (*n* = 37), golimumab (*n* = 8), or certolizumab (*n* = 16)] according to national recommendations and followed-up for 6 months (14). Forty-five patients were from La Paz University Hospital and 33 patients from Ramón y Cajal University Hospital, in Madrid, Spain. All included patients were adults (age over 18), fulfilled the ACR/EULAR 2010 classification criteria for RA, and had moderate or high disease activity (DAS28 > 3.2) (15). Approvals were obtained from the Institutional Ethics Committees from both centers (PI-018/17; PI-2618) in accordance with the Helsinki Declaration. All patients signed an informed consent document before inclusion.

### Clinical Data

Disease activity was assessed by DAS28 at baseline (before starting TNFi) and after 6 months of treatment. REM was defined as DAS28 ≤ 2.6 after 6 months of TNFi therapy. The achievement of REM was established as our clinical outcome in responder patients (16). In addition, disease activity was assessed by the Simple Disease Activity Index (SDAI), and REM was defined as SDAI ≤ 3.3 for the sensitivity analysis (17).

### Isolation of PBMC From Human Peripheral Blood

Blood samples were collected at baseline and after 6 months of TNFi treatment. For baseline samples, blood was collected from patients just before starting TNFi. For samples at 6 months, blood was collected within 24 h before TNFi administration. PBMC were purified from heparinized venous blood by Ficoll®-Paque PREMIUM (GE Healthcare, Chicago, IL, USA) density gradient centrifugation, and cells were subsequently cryopreserved (10% DMSO) in liquid N_2_ in aliquots of 5 × 10^6^ cells until studied (18). Basal and 6-month samples were studied simultaneously to avoid interassay variability. We recorded for every leukocyte subset total cell counts per ml of blood calculated by measuring total lymphocyte and monocyte numbers by a Coulter counter and the percentages of every subset over total mononuclear cells.

### Flow Cytometry Analysis

PBMC were thawed and resuspended (10^6^ cells/ml) in RPMI 1,640 medium (Life Technologies, Carlsbad, CA, USA) supplemented with 10% heat-inactivated fetal bovine serum (Life Technologies), 1% L-glutamine, and 1% penicillin–streptomycin at the time of analysis. PBMC were subsequently incubated with specific monoclonal antibodies for membrane antigen staining during 20 min at 4°C in the dark and then washed with PBS. Then, PBMC were acquired on a FACSCanto II cytometer (BD Biosciences, San Diego, CA, USA). Data were analyzed using FACSDiva software, version 8.0.1 (BD Biosciences). Mean autofluorescence values were set using appropriate negative isotype controls.

Monocyte and lymphocyte populations were studied. The following monoclonal antibodies were used: CD4-FITC, CD14-FITC, CD197-PE (CCR7-PE), CD3-PerCP, CD19-PE-Cy7, CD56-PE-Cy7, CD45RO-APC, CD27-APC, CD8-APC-H7, CD3-BV421, and CD45-V500-C (all from BD Biosciences). According to the differential expression of several antigens, CD4^+^ and CD8^+^ T cells were classified as naive (CCR7^+^ CD45RO^−^), central memory (CCR7^+^ CD45RO^+^), effector memory (CCR7^−^ CD45RO^+^), or terminally differentiated effector memory (CCR7^−^ CD45RO^−^). B cells were classified as total CD19^+^ B cells, naive (CD19^+^ CD27^−^), or memory (CD19^+^ CD27^+^). CD56^+^ cells were subdivided into NK cells (CD56^dim^ CD3^−^), natural killer T (NKT) cells (CD56^dim^ CD3^+^), and NK regulatory cells (CD3^−^ CD56^bright^). Monocytes were classified as CD14^+^ cells. Gating strategies are described in Supplementary Figures 1, 2. A gate including lymphocytes and monocytes, but excluding debris, duplets, and apoptotic cells, was established. Viable CD45^+^ cells were confirmed by nonstaining with 7-AAD (Supplementary Figure 1, gate R6). A minimum amount of 100,000 events concerning viable CD45^+^ cells was analyzed.

### Statistical Analyses

Descriptive analyses were performed for the demographic and clinical variables. The results were shown as mean and SD [or median and interquartile range (IQR)] for continuous variables and absolute numbers and relative frequencies for categorical variables. The frequency data were compared using Fisher's exact tests. Comparisons of unpaired continuous data were conducted using the unpaired *t*-test or Mann–Whitney *U*-test, depending on data distribution. Comparisons of paired continuous data were conducted using the paired *t*-test or Wilcoxon, depending on data distribution. In a previous study conducted by our group including the same cohort of patients, the correction of multiple comparisons using the Benjamini–Hochberg (BH) method with a predefined value FDR = 0.25 was applied (free software used MEV 2.0). This analysis demonstrated that the associations of either total or naive B lymphocytes with REM were not due to chance (13). Therefore, clinical follow-up analyses after initiation of TNFi therapy have been performed in these two subpopulations.

The associations between either the clinical/serological variables or the percentage of change (Δ, 6–0 months) within each PBMC subset and REM at 6 months were evaluated by uni- and multivariable logistic regression (odds ratio; 95% CI). The presence of possible interactions with covariates was tested, stratifying the results if significant. In case of no significant interaction, the model was later adjusted for these covariates.

A *p*-value < 0.05 was considered as statistically significant. The Statistical Package for the Social Sciences version 24 (SPSS, Chicago, IL, USA) was used for the analyses. The GraphPad Prism version 7 (GraphPad Software, San Diego, CA, USA) was used to prepare the graphs.

### Patient and Public Involvement

Patients did not cooperate with us in the design of the study. However, it was explained to them prior to inclusion, and they all agreed to participate.

## Results

### Patients' Characteristics

Demographic and clinical baseline characteristics are shown in Table 1. The mean baseline disease activity (DAS28) was 4.9. Forty-one (53%) patients were treated with TNFi monoclonal antibodies (infliximab, adalimumab, golimumab, certolizumab) and 37 (47%) patients with a TNFi fusion protein (etanercept). Concomitant MTX was administered in 58 (74%) patients. After 6 months of TNFi therapy, 29 (37%) patients achieved REM by DAS28. According to clinical REM achievement (DAS28 ≤ 2.6) after 6 months of TNFi treatment, differences at baseline between REM and non-REM patients were analyzed. Overall, no differences between baseline characteristics in both groups were found, except for DAS28. Baseline DAS28 was lower in patients who attained REM (4.2 ± 0.9 vs. 5.3 ± 0.9; *p* < 0.001).

**Table 1:** Baseline characteristics of patients included in the study.

**Baseline patients' characteristics**	**Total patients (*n* = 78)**	**Non-REM (*n* = 49; 63%)**	**REM (*n* = 29; 37%)**	***p*-value**
Age (years)	54 ± 12	55 ± 13	51 ± 11	0.1
Female	67 (86)	43 (88)	24 (83)	0.5
Disease duration (years)	6 (3, 11)	8 (4, 12)	6 (3, 10)	0.7
RF positive	60 (77)	35 (71)	25 (86)	0.1
ACPA positive	65 (83)	38 (78)	27 (93)	0.07
Smoking habit (*n* = 64)				0.4
Never smokers	29 (45)	19 (50)	10(38)	
Smoker (past or current)	35 (55)	19 (50)	16 (62)	
Body mass index (kg/m2)	25.4 (22.8–30.2)	25.6 (23.0–30.2)	24.7 (22.4–30.4)	0.6
DAS28	4.9 ± 1.0	5.3 ± 0.9	4.2 ± 0.9	**<0.001**
Type of TNFi				0.1
Monoclonal antibody	41 (53)	29 (59)	12 (41)	
Etanercept	37 (47)	20 (41)	17 (59)	
Concomitant csDMARDs	75 (96)	48 (98)	27 (93)	0.7
MTX (± OD)	58 (74)	39 (80)	19 (68)	0.2
Only OD	17 (22)	9 (18)	8 (25)	0.2
Prednisone	45 (58)	30 (61)	15 (54)	0.5

*The table shows mean ± SD, median (IQR), or absolute number (percentage) for the patients included (n = 78). The results are also stratified by remission status after 6 months. The frequency data were compared using Fisher's exact tests. Comparisons of continuous data were conducted using the unpaired t-test or Mann–Whitney U-test, depending on the data distribution. Significant statistical differences are noted in bold. p-value <0.05 was considered statistically significant. RF, rheumatoid factor; ACPA, anti-citrullinated peptide antibody; csDMARDs, conventional synthetic disease-modifying antirheumatic drug; DAS28, Disease Activity Score-28; TNFi, tumor necrosis factor inhibitor; MTX, methotrexate; OD, other csDMARDs*.

Univariable analyses were performed to investigate the association between REM and the baseline patients' characteristics. A significant association was found for lower baseline DAS28 (OR: 0.28; 95% CI: 0.19–0.51). Associations of REM with other baseline patients' characteristics, such as age (OR: 0.97; 95% CI: 0.94–1.01), rheumatoid factor (RF) positivity (OR: 2.50; 95% CI: 0.73–8.50), anti-citrullinated protein antibody (ACPA) positivity (OR: 3.91; 95% CI: 0.80–19.07), or the type of TNFi (reference fusion protein, etanercept) used (OR: 2.05; 95% CI: 0.81–5.22) were also found, although they were not significant (Table 2). Therefore, further analyses were adjusted by the patients' and disease's characteristics with a *p* < 0.1 in the univariable analysis (ACPA and baseline DAS28) as well by the type of TNFi used.

**Table 2:** Association between patients' characteristics and clinical remission (DAS28 ≤ 2.6) after 6 months of TNFi treatment.

**Baseline patients' characteristics**	**OR**	**95% CI**	***p*-value**
Sex (female)	0.67	0.18–2.42	0.5
Age (years)	0.97	0.94–1.01	0.1
Disease duration (years)	1.00	0.97–1.02	0.7
RF positive	2.50	0.73–8.50	0.1
ACPA positive	3.91	0.80–19.07	0.09
Smokers	1.60	0.58–4.41	0.4
Body mass index (kg/m^2^)	0.97	0.88–1.07	0.6
Baseline DAS28	0.28	0.19–0.51	**<0.001**
Type of TNFi (etanercept)	2.05	0.81–5.22	0.1
Concomitant MTX	0.54	0.19–1.55	0.2
Prednisone	0.73	0.29–1.87	0.5
Δ (total B cell)	0.76	0.61–0.94	**0.01**
Δ (naive B cell)	0.85	0.73–0.99	**0.04**
Δ (memory B cell)	0.96	0.77–1.19	0.7

*Associations were evaluated by univariable logistic regression. Odds ratio (OR) and 95% confidence interval (CI) were calculated. Significant statistical differences are noted in bold. p-value < 0.05 was considered statistically significant. RF, rheumatoid factor; ACPA, anti-citrullinated peptide antibody; csDMARDs, conventional synthetic disease-modifying antirheumatic drug; DAS28, Disease Activity Score-28; TNFi, tumor necrosis factor inhibitor; MTX, methotrexate*.

### PBMC Subset Changes After 6 Months of TNFi Treatment

Different PBMC subsets were analyzed at baseline and after 6 months of TNFi treatment, in order to identify whether PBMC profile changes after TNFi therapy (Δ, 6–0 months). A previous analysis in our cohort of patients showed that a higher percentage of baseline B cells (especially naive B cells) was associated with attaining REM after 6 months of TNFi treatment (13).

The present results showed a significant reduction in the percentage of total B cells in patients who achieved REM compared with non-REM patients, after 6 months of TNFi treatment (*p* = 0.01). It was mainly due to a reduction of naive B cells in REM patients (*p* = 0.04). No differences in percentage for any other PBMC subset analyzed were observed (Supplementary Table 1). In addition, the analysis of the absolute cell number was performed; however, no differences were found for none of the PBMC subset evaluated (Supplementary Table 4).

### Association Between PBMC Subset Changes and Clinical Remission

In The Univariable analysis, a significant association between REM achievement and a reduction of total B-cell percentage (OR: 0.76; 95% CI: 0.61–0.94) was found. After evaluating B-cell subtypes, it was observed that this association was mainly due to a reduction in naive B cells (OR: 0.85; 95% CI: 0.73–0.99) (Table 2). No significant associations were found for the other analyzed PBMC subsets (Supplementary Table 2).

The presence of interactions between the change of naive B cells (Δ naive B cells) and REM achievement after 6 months of TNFi therapy was tested. A significant interaction between the change in the percentage of naive B cells and the use of concomitant MTX was found (Wald chi-square value = 4.56; *p* = 0.03). Therefore, further analyses over the association between the Δ naive B cells and REM were stratified according to the use of concomitant MTX. No significant interactions with other variables were found (Supplementary Table 3).

Fifty-eight (74%) patients received concomitant MTX. Interestingly, the results of the multivariable analysis showed that a reduction in the percentage of naive B cell was independently associated with REM after 6 months of treatment with TNFi only in the group of patients who received concomitant MTX (OR: 0.59; 95% CI: 0.39–0.91). No association was found for the group of patients who did not receive concomitant MTX (OR: 0.85; 95% CI: 0.63–1.16). However, a lower DAS28 at baseline was also independently associated with REM, regardless of the use of concomitant MTX (Table 3).

**Table 3:** Analysis of the association between Δ (naive B cell) and clinical remission (DAS28 ≤ 2.6) after 6 months of TNFi treatment, stratified by the concomitant use of MTX.

**Patients' characteristics**	**No-MTX use**			**MTX use**		
	**OR**	**95% CI**	***p*-value**	**OR**	**95% CI**	***p*-value**
Baseline DAS28	0.17	0.03–0.94	**0.04**	0.21	0.08–0.51	**<0.001**
ACPA positive	6.33	0.30–135.08	0.2	2.76	0.26–29.75	0.4
Type of TNFi (etanercept)	1.06	0.09–12.15	1.0	3.62	0.71–18.37	0.1
Δ (naive B cell)	0.85	0.63–1.16	0.3	0.59	0.39–0.91	**0.01**

*Logistic regression analysis adjusted by baseline DAS28 and ACPA seropositivity. Odds ratio (OR) and 95% confidence interval (CI) and p-values were calculated. Significant statistical differences are noted in bold. p-value < 0.05 was considered as statistically significant. DAS28, Disease Activity Score-28; ACPA, anti-citrullinated peptide antibody; TNFi, tumor necrosis factor inhibitor*.

In order to confirm our results, a sensitivity analysis was performed employing the SDAI definition for clinical REM (SDAI ≤ 3.3). Out of the 78 patients, 17% (*n* = 13) achieved clinical remission according to this criterion. The results were consistent with the evaluation through DAS28, finding that a reduction in the percentage of naive B cells was associated with REM after 6 months of treatment with TNFi, mainly in the group of patients who received concomitant MTX (OR: 0.45; 95% CI: 0.24–0.88). However, no association was found for the group of patients who did not receive concomitant MTX (OR: 0.84; 95% CI: 0.66–1.08).

## Discussion

In this study, we aimed to investigate how peripheral blood cell profile can be modified after TNFi treatment, according to the clinical response in patients with RA. The results showed that a reduction in the percentage of peripheral naive B cells was associated with attaining REM in patients with RA treated with TNFi, mainly in combination with MTX.

The latest breakthroughs in the physiopathology of RA highlighted the activation of naive B cells as the trigger of the joint flare initiation and, therefore, gave them a central role in the pathogenesis of the disease (19, 20). Although the mechanism of this B-cell activation remains unknown, several investigations have pointed out that defects on B-cell receptor (BCR) signaling may cause breakdown of B-cell tolerance. This may lead to maturation of autoreactive B cells, associated to the development of autoimmunity (21, 22) and to the subsequent RF and ACPA production (23–25). Moreover, it has been demonstrated that activated B cells can act as antigen-presenting cells by the processing and presenting of antigenic peptides to CD4^+^ T cells (26). This presentation ability promotes or triggers the proinflammatory environment that is found in RA (27). Furthermore, this B-cell ability would be enhanced in RF-positive patients, given that B cells can capture antigen–Ig immune complexes *via* their membrane immunoglobulin receptors and react with the IgG Fc part of antibody molecules. Then, antigen is processed and presented to CD4^+^ T cells, initiating the immune response (28).

Although it is still unclear and controversial, some publications have suggested that B cells could play a relevant role in the response to TNFi. This controversy may be related to the variability within the different RA cohorts (age, disease duration, seropositivity, disease activity, treatment with TNFi and/or csDMARDs) and the different criteria used to assess clinical response (10, 29). These cofounders should be taken into account when differences between cell populations are analyzed. Moura et al. suggested, in a study with a small number of patients, that treatment with TNFi restores the frequency of a subtype of peripheral memory B cells to normal levels, regardless of the clinical response (12). Moreover, Daien et al. reported that a high baseline level of CD27^+^ memory B cells was associated with good clinical response to TNFi (10). In addition, a different B-cell subpopulation that could be affected by TNFi treatment is IL-10-producing memory B cells (B10). Bankó et al. suggested that B10 increases after TNFi treatment in responding RA patients (9). In our study, in which all the analyses were adjusted by confounders, a significant decrease in the percentage of B cells in REM patients after 6 months of TNFi therapy, mainly due to naive B cells, was found. These results could suggest that this reduction is related to the fact that patients in REM had lower production of cytokines such as interleukin-6 (IL-6), TNFα, interleukin-12 (IL-12), or granulocyte macrophage-colony stimulating factor (GM-CSF), by different PBMC subsets (30, 31). In consequence, it is hypothesized that the reduction in the frequency of naive B cells in patients who achieved remission is mainly due to the improvement of inflammatory activity, regardless of the type of drug itself. To confirm this hypothesis, it would be highly interesting to evaluate it with other bDMARDs, or even with targeted synthetic DMARDs or csDMARDs.

A novel and interesting finding of our study is that the association between the reduction in the percentage of naive B cells and the achievement of REM was mainly observed in the group of patients who received TNFi concomitantly with MTX. Therefore, because of MTX mechanism of action, T cells would reduce the production of cytokines such as TNFα, IL-6, or GM-CSF (32). The diminished IL-6 and GM-CSF production may have as a consequence lesser development and survival of B cells (33, 34). Consequently, their reduction by MTX may be impairing the development of B cells (35). A recent study from our research group reported that patients with early RA who responded to MTX therapy had significantly decreased circulating transitional B cells, supporting also the impairment of B-cell development (36). Although the effect of MTX would seem enough potent to improve the clinical manifestations of the disease, our cohort showed that the proportion of patients using concomitant MTX was no different between REM and non-REM groups. In this sense, it is relevant to take into account that the activity indices to control RA include both inflammatory (swollen joints and acute phase reactants) and others less dependent on inflammation (tender joints and patient global assessment) parameters. Indeed, there may be patients in clinical REM who persist having tender and/or swollen joints, as well as elevation of acute phase reactants. It would be very interesting to conduct further studies to investigate whether the co-administration of TNFi and MTX produces a synergistic effect on the immunological expression of inflammatory molecules that translates into greater clinical remission of the disease.

Currently, the core principles of the *treat-to-target (T2T) strategy* in RA are shared decision-making and regular patient review with a target of REM or, failing that, low disease activity to ensure optimal outcomes (37, 38). Therefore, objective parameters of REM are necessary to achieve a successful outcome following the T2T strategy. In this study, we identify that naive B-cell subset is modulated after TNFi therapy in patients who achieve REM. At the moment, we do not know whether this may have implications with long-term outcome measurements and therefore could be useful to monitor TNFi therapy. In addition, independent to the reduction in the percentage of naive B cells after 6 months of TNFi treatment, a low baseline DAS28 was also associated with REM achievement. This result was in agreement with previous investigations. Aletaha et al. demonstrated that lower baseline disease activity is associated with achieving clinical REM (39).

One of the limitations of our study was the population included; however, sample size calculation (before starting the patient recruitment) showed that 78 patients naive to TNFi would be enough to demonstrate statistical differences in the proposed clinical outcomes. The other limitation of the study was that patients who attained REM after 6 months of TNFi treatment showed lower baseline DAS28 than patients who did not attain it. However, baseline disease activity did not interact with the association between the reduction of the percentage of naive B cells and REM, both parameters remaining independently associated. On the other hand, as it was previously described (12), CD19^+^ CD27^−^ cells are mostly naive B cells, but a small proportion of them are memory B cells. However, due to our gating strategy that did not include IgD, it was not possible to discriminate this small subset of double negative memory B cells defined as CD19^+^ CD27^−^ IgD^−^. Another limitation of our study was not performing *in vitro* functional evaluation to analyze in detail the mechanism of TNFi (± MTX) in reducing the percentage of naive B cells. Finally, another interesting aspect to consider in future studies would be to add a group with only MTX and evaluate if similar changes on naive B cells occur in patients who achieved clinical remission.

Our future research agenda, following this line of work, will include an in-depth study into this research field through evaluation of differences in the intracellular cytokine production that could help to better understand the changes in PBMC, according to the clinical response.

In conclusion, our study suggests that PBMC (principally the B-cell subsets) are modulated in patients with RA in clinical REM after 6 months of TNFi therapy. A significant decrease in naive B-cell percentage is associated with achieving REM, mainly in patients who received concomitant MTX. However, further research studies with other cohorts of patients need to be performed before implementing the results of this study.

## Data Availability Statement

The original contributions presented in the study are included in the article/Supplementary Material, further inquiries can be directed to the corresponding authors.

## Ethics Statement

The studies involving human participants were reviewed and approved by Ethics committees of Ramón y Cajal (PI-018/17) and La Paz University Hospitals (PI-2618), Madrid. The patients/participants provided their written informed consent to participate in this study.

## Author Contributions

CP-R, LV, DP-S, and ER-M planned the study. BH-B, CG-H, IN-G, TJ, and PL-S collected the samples and performed flow cytometry experiments. BH-B wrote the manuscript draft. ER-M and LV supervised flow cytometry studies. BH-B, CP-R, VN-C, and MD-A performed and supervised statistical studies. CP-R, VN-C, CS, JB-C, GB, CP-M, MV-D, and AB visited RA patients and collected clinical data. ER-M and CP-R had full access to all of the data in the study and take responsibility for the integrity of the data and the accuracy of the data analysis. All authors were involved in revising the manuscript critically for important intellectual content, approved the final version to be published, and contributed to the manuscript and approved the submitted version.

## Conflict of Interest

CP-R has received research grants/honoraria from AbbVie, Lilly, Novartis, Pfizer, Sanofi, Biogen and UCB. VN-C reports speaker fees and grants from Abbvie, Janssen, Lilly, MSD, Novartis, Pfizer and UCB during the conduct of the study. AB reports grants, consultancies and speaker fees from Abbvie, BMS, Nordic, Novartis, Pfizer, Sandoz, Sanofi, Roche and UCB during the conduct of the study. DP-S reports speaker fees and grants from Abbvie, Grifols, Menarini, Novartis, Pfizer and Takeda during the conduct of the study. The remaining authors declare that the research was conducted in the absence of any commercial or financial relationships that could be construed as a potential conflict of interest.
