# Supplementary figures and images for: Remission Induced by TNF Inhibitors Plus Methotrexate is Associated With Changes in Peripheral Naïve B Cells in Patients With Rheumatoid Arthritis
Source: Front Med (Lausanne). 2021 Jun 17;8:683990. doi: 10.3389/fmed.2021.683990 (PMC8245775; doi:10.3389/fmed.2021.683990)

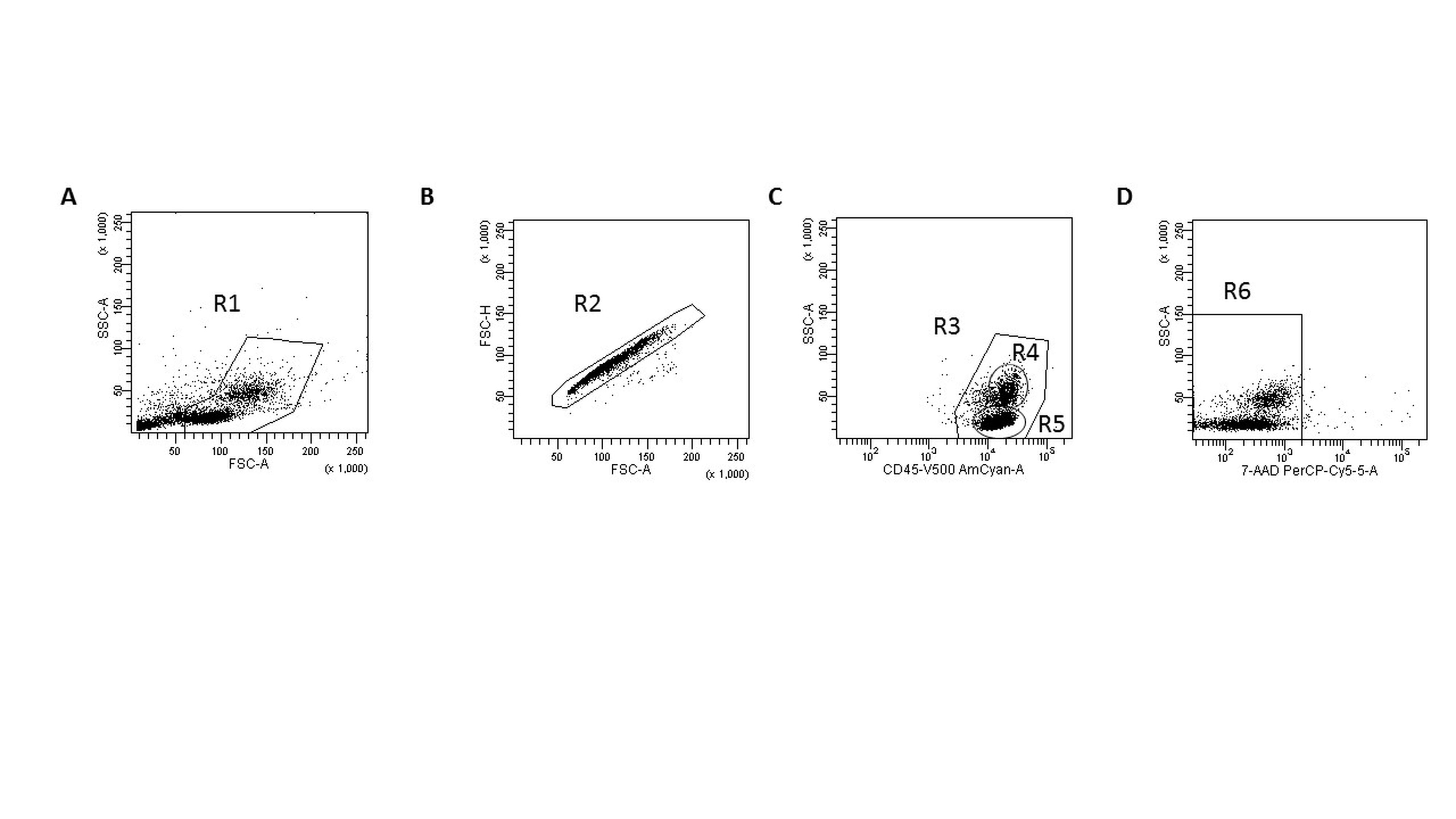

Supplement: Supplementary file 1 [file Image_1.JPEG]

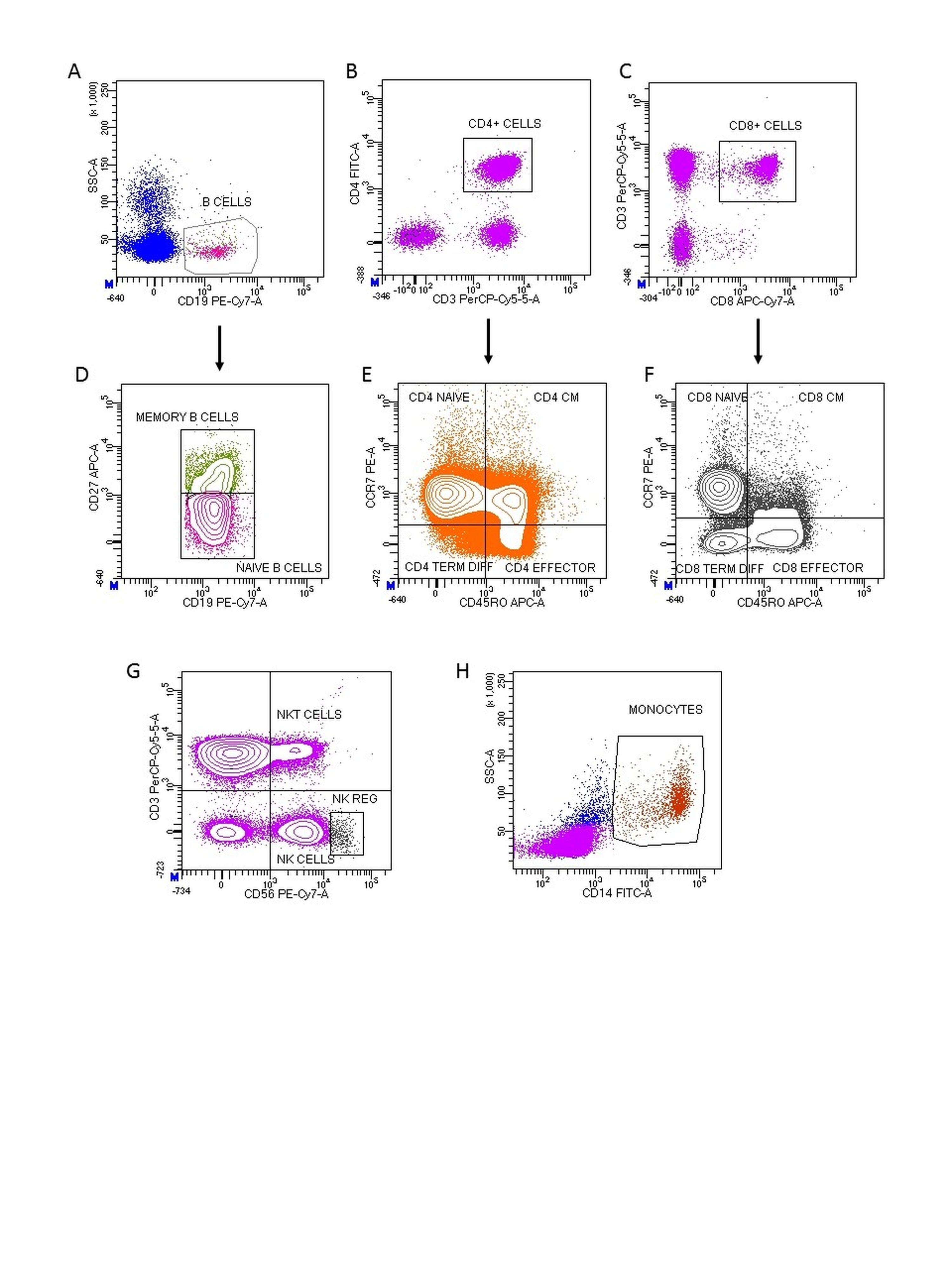

Supplement: Supplementary file 2 [file Image_2.JPEG]
