# Supplementary material for: Remission Induced by TNF Inhibitors Plus Methotrexate is Associated With Changes in Peripheral Naïve B Cells in Patients With Rheumatoid Arthritis
Source: Front Med (Lausanne). 2021 Jun 17;8:683990. doi: 10.3389/fmed.2021.683990 (PMC8245775; doi:10.3389/fmed.2021.683990)
Supplement: Supplementary file 3 [file Table_1.DOCX]

**Table S1: PBMC subsets at baseline, at 6m and the change Δ (6m-0m) during the TNFi treatment according to clinical remission.** PBMC subsets measured by flow cytometry in percentage (related to CD45^+^ cells). Results are expressed as median and interquartile range. Depending on data distribution, unpaired t or Mann-Whitney U test were employed to analyse the differences between non-REM and REM patients. P-value<0.05 was considered as statistically significant. Significant differences are noted in bold.

| **% (ref: mononuclear cells)** | **baseline** | | | **6 months** | | | **∆ (6m-0m)** | | |
| --- | --- | --- | --- | --- | --- | --- | --- | --- | --- |
| **PBMC subset** | **Non-REM**  **(n=49)** | **REM**  **(n=29)** | **P-value** | **Non-REM**  **(n=49)** | **REM**  **(n=29)** | **P-value** | **Non-REM**  **(n=49)** | **REM**  **(n=29)** | **P-value** |
| **Monocytes (CD14^+^)** | 6.9 (3.8-10.9) | 8.4 (5.5-13.6) | 0.1 | 7.1 (4.4-9.9) | 12.0 (2.9-15.0) | **0.04** | -0.2 [(-2.9)-2.2] | -0.7 [(-3.5)-6.4] | 0.8 |
| **NK cells (CD56^dim^ CD3^-^)** | 13.0 (7.2-16.4) | 9.5 (5.7-14.0) | 0.6 | 14.4 (8.3-19.5) | 12.7 (7.9-17.3) | 1.0 | 0.8 [(-1.8)-3.2] | 2.5 [(-0.7)-4.9] | 0.1 |
| **NKT cells (CD56^dim^ CD3^+^)** | 3.6 (2.0-6.4) | 4.7 (2.9-8.1) | 0.1 | 3.8 (2.3-7.9) | 5.3 (3.6-7.9) | 0.1 | 0.2 [(-0.5)-1.4] | 0.3 [(-1.5)-1.9] | 1.0 |
| **NK bright cells (CD3^-^ CD56^bright^)** | 0.6 (0.3-1.1) | 0.6 (0.3-1.3) | 0.7 | 0.8 (0.3-1.3) | 0.8 (0.5-1.4) | 0.3 | -0.0 [(-0.1)-0.2] | 0.2 [(-0.1)-0.5] | 0.3 |
| **Total CD4^+^ T cells** | 46.3 (34.7-57.2) | 43.5 (26.1-55.0) | 0.3 | 42.9 (31.6-52.6) | 45.9 (30.9-53.4) | 1.0 | -3.5 [(-7.4)-3.9] | -0.4 [(-9.1)-7.2] | 0.2 |
| **Naïve CD4^+^ T cells (CCR7^+^ CD45RO^-^)** | 22.2 (8.8-34.0) | 20.4 (8.2-30.4) | 0.6 | 18.2 (9.6-26.7) | 20.5 (12.2-24.6) | 0.8 | -1.2 [(-6.8)-1.9] | -1.3 [(-8.8)-1.9] | 0.7 |
| **Central memory CD4^+^ T cells (CCR7^+^ CD45RO^+^)** | 14.1 (11.7-17.7) | 14.1 (10.7-18.8) | 0.8 | 14.5 (10.2-18.4) | 14.6 (10.9-21.3) | 0.5 | -0.5 [(-1.9)-1.9] | 1.1 [(-0.8)-4.3] | 0.2 |
| **Effector memory CD4^+^ T cells (CCR7^-^ CD45RO^+^)** | 3.6 (1.3-6.5) | 5.5 (2.8-7.5) | 0.9 | 4.1 (1.3-7.8) | 5.0 (2.9-8.5) | 1.0 | 0.1 [(-0.5)-1.5] | 0.0 [(-0.7)-1.0] | 0.6 |
| **Terminally differentiated CD4^+^ T cells (CCR7^-^ CD45RO^-^)** | 2.0 (0.8-4.6) | 2.7 (0.5-6.4) | 0.8 | 1.9 (0.7-4.2) | 2.8 (0.6-5.5) | 0.9 | 0.0 [(-0.5)-0.1] | 0.2 [(-0.4)-1.3] | 0.1 |
| **Total CD8^+^ T cells** | 11.9 (8.2-17.7) | 14.4 (12.3-18.0) | 0.09 | 12.8 (8.7-17.3) | 15.2 (11.3-16.6) | 0.2 | 0.2 [(-1.9)-1.6] | -0.6 [(-2.2)-1.8] | 0.6 |
| **Naïve CD8^+^ T cells (CCR7^+^ CD45RO^-^)** | 3.5 (1.8-5.7) | 4.1 (2.6-7.0) | 0.2 | 3.2 (2.1-5.7) | 3.8 (2.2-6.1) | 0.6 | -0.3 [(-1.0)-0.6] | -0.5 [(-1.9)-0.3] | 0.1 |
| **Central memory CD8^+^ T cells (CCR7^+^ CD45RO^+^)** | 1.1 (0.8-1.7) | 1.2 (0.9-1.8) | 0.7 | 1.1 (0.8-1.9) | 1.4 (0.8-1.9) | 0.7 | -0.1 [(-0.3)-0.2] | 0.0 [(-0.4)-0.4] | 0.9 |
| **Effector memory CD8^+^ T cells (CCR7^-^ CD45RO^+^)** | 2.2 (1.1-4.1) | 2.7 (1.4-4.4) | 0.4 | 2.3 (1.0-5.0) | 2.4 (1.6-4.1) | 0.5 | 0.1 [(-0.4)-1.0] | 0.1 [(-0.6)-0.8] | 0.3 |
| **Terminally differentiated CD8^+^ T cells (CCR7^-^ CD45RO^-^)** | 3.1 (1.9-5.6) | 3.0 (1.8-5.6) | 0.7 | 3.4 (2.0-5.4) | 3.3 (1.9-8.2) | 0.2 | 0.0 [(-0.9)-0.8] | 0.1 [(-0.7)-1.4] | 0.3 |
| **Total B cells (CD19^+^)** | 4.1 (3.0-7.3) | 5.8 (3.9-8.8) | **0.02** | 4.2 (2.8-7.5) | 5.1 (3.6-6.6) | 0.3 | -0.3 [(-1.3)-0.8] | -1.0 [(-3.1)-0.4] | **0.01** |
| **Naïve B cells (CD19^+^ CD27^-^)** | 3.5 (2.1-6.1) | 4.4 (2.9-7.0) | **0.04** | 4.2 (2.4-6.2) | 4.3 (3.0-5.7) | 0.3 | 0.2 [(-0.7)-1.3] | -0.4 [(-3.3)-0.8] | **0.04** |
| **Memory B cells (CD19^+^ CD27^+^)** | 0.9 (0.6-1.3) | 1.1 (0.7-1.5) | 0.07 | 1.1 (0.6-1.4) | 1.1 (0.7-1.8) | 0.4 | 0.1 [(-0.1)-0.4] | -0.1 [(-0.5)-0.2] | 0.7 |
