# Supplementary material for: Remission Induced by TNF Inhibitors Plus Methotrexate is Associated With Changes in Peripheral Naïve B Cells in Patients With Rheumatoid Arthritis
Source: Front Med (Lausanne). 2021 Jun 17;8:683990. doi: 10.3389/fmed.2021.683990 (PMC8245775; doi:10.3389/fmed.2021.683990)
Supplement: Supplementary file 4 [file Table_2.DOCX]

**Table S2: Association between the percentage of change within each PBMC subset and clinical remission (DAS28≤2.6) after 6 months of TNFi treatment.** Logistic regression analysis. Odds ratio (OR) and 95% confidence interval (CI) and p-values were calculated. Significant statistical differences are noted in bold. P-value<0.05 was considered as statistically significant.

| **PBMC subset** | **OR** | **95% CI** | **P-value** |
| --- | --- | --- | --- |
| **Monocytes (CD14^+^)** | 1.05 | 0.97-1.13 | 0.2 |
| **NK cells (CD56^dim^ CD3^-^)** | 1.03 | 0.95-1.12 | 0.4 |
| **NKT cells (CD56^dim^ CD3^+^)** | 1.00 | 0.83-1.20 | 1.0 |
| **NK regulatory cells (CD3^-^ CD56^bright^)** | 1.00 | 0.55-1.82 | 1.0 |
| **Total CD4^+^ T cells** | 1.03 | 0.99-1.08 | 0.2 |
| **Naïve CD4^+^ T cells (CCR7^+^ CD45RO^-^)** | 1.01 | 0.95-1.08 | 0.7 |
| **Central memory CD4^+^ T cells (CCR7^+^ CD45RO^+^)** | 1.08 | 0.96-1.20 | 0.2 |
| **Effector memory CD4^+^ T cells (CCR7^-^ CD45RO^+^)** | 0.94 | 0.73-1.21 | 0.6 |
| **Terminally differentiated CD4^+^ T cells (CCR7^-^ CD45RO^-^)** | 1.23 | 0.93-1.61 | 0.2 |
| **Total CD8^+^ T cells** | 0.97 | 0.87-1.08 | 0.6 |
| **Naïve CD8^+^ T cells (CCR7^+^ CD45RO^-^)** | 0.79 | 0.57-1.09 | 0.1 |
| **Central memory CD8^+^ T cells (CCR7^+^ CD45RO^+^)** | 0.97 | 0.49-1.91 | 0.9 |
| **Effector memory CD8^+^ T cells (CCR7^-^ CD45RO^+^)** | 0.83 | 0.58-1.17 | 0.3 |
| **Terminally differentiated CD8^+^ T cells (CCR7^-^ CD45RO^-^)** | 1.03 | 0.88-1.21 | 0.9 |
| **Total B cells (CD19^+^)** | 0.76 | 0.61-0.94 | **0.01** |
| **Naïve B cells (CD19^+^ CD27^-^)** | 0.85 | 0.73-0.99 | **0.04** |
| **Memory B cells (CD19^+^ CD27^+^)** | 0.96 | 0.77-1.19 | 0.7 |
