# Supplementary material for: Remission Induced by TNF Inhibitors Plus Methotrexate is Associated With Changes in Peripheral Naïve B Cells in Patients With Rheumatoid Arthritis
Source: Front Med (Lausanne). 2021 Jun 17;8:683990. doi: 10.3389/fmed.2021.683990 (PMC8245775; doi:10.3389/fmed.2021.683990)
Supplement: Supplementary file 5 [file Table_3.docx]

**Table S3: Analysis of interactions between ∆ (naïve B cell) with clinical remission (DAS28≤2.6) after 6 months of TNFi treatment.** Significant statistical differences are noted in bold. P-value<0.05 was considered as statistically significant. RF, rheumatoid factor; ACPA, anti-citrullinated peptide antibody; DAS28, disease activity score-28; TNFi, tumor necrosis factor inhibitor; MTX, methotrexate.

| **Baseline patients’ characteristics** | **Wald chi-square** | **p-value** |
| --- | --- | --- |
| **Sex (female)** | 0.98 | 0.3 |
| **Age (years)** | 1.85 | 0.2 |
| **Disease duration (years)** | 1.46 | 0.2 |
| **RF positive** | 2.51 | 0.1 |
| **ACPA positive** | 1.93 | 0.2 |
| **Smokers** | 2.93 | 0.1 |
| **Body mass index (kg/m^2^)** | 0.01 | 0.9 |
| **Baseline DAS28** | 0.01 | 0.9 |
| **Type of TNFi (etanercept)** | 0.01 | 0.9 |
| **Concomitant MTX** | 4.56 | **0.03** |
| **Prednisone** | 2.42 | 0.1 |
