# Supplementary material for: Remission Induced by TNF Inhibitors Plus Methotrexate is Associated With Changes in Peripheral Naïve B Cells in Patients With Rheumatoid Arthritis
Source: Front Med (Lausanne). 2021 Jun 17;8:683990. doi: 10.3389/fmed.2021.683990 (PMC8245775; doi:10.3389/fmed.2021.683990)
Supplement: Supplementary file 6 [file Table_4.DOC]

| **Absolute cell count (cells/µL)** | **Baseline** | | | **6 months** | | | **∆ (6m-0m)** | | |
| --- | --- | --- | --- | --- | --- | --- | --- | --- | --- |
| **PBMC subset** | **Non-REM**  **(n=49)** | **REM**  **(n=29)** | **P-value** | **Non-REM**  **(n=49)** | **REM**  **(n=29)** | **P-value** | **Non-REM**  **(n=49)** | **REM**  **(n=29)** | **P-value** |
| **Monocytes (CD14^+^)** | 194±161 | 193±140 | 1.0 | 394±387 | 471±419 | 0.4 | 209±325 | 303±378 | 0.3 |
| **NK cells (CD56^dim^ CD3^-^)** | 306±312 | 224±177 | 0.07 | 742±566 | 665±525 | 0.6 | 454±449 | 461±384 | 0.9 |
| **NKT cells (CD56^dim^ CD3^+^)** | 125±139 | 117±84 | 0.8 | 340±439 | 316±286 | 0.8 | 224±322 | 216±246 | 0.9 |
| **NK bright cells (CD3^-^ CD56^bright^)** | 20±21 | 18±17 | 0.7 | 56±65 | 55±54 | 1.0 | 38±54 | 41±45 | 0.8 |
| **Total CD4^+^ T cells** | 1062±517 | 816±444 | **0.04** | 2007±1077 | 1822±958 | 0.4 | 981±1041 | 1092±955 | 0.7 |
| **Naïve CD4^+^ T cells (CCR7^+^ CD45RO^-^)** | 520±405 | 402±314 | 0.2 | 838±572 | 736±406 | 0.4 | 332±469 | 372±415 | 0.7 |
| **Central memory CD4^+^ T cells (CCR7^+^ CD45RO^+^)** | 346±177 | 284±127 | 0.1 | 807±622 | 772±514 | 0.8 | 479±594 | 531±460 | 0.7 |
| **Effector memory CD4^+^ T cells (CCR7^-^ CD45RO^+^)** | 121±122 | 104±69 | 0.5 | 288±439 | 242±205 | 0.6 | 0±2 | 0±1 | 0.6 |
| **Terminally differentiated CD4^+^ T cells (CCR7^-^ CD45RO^-^)** | 72±67 | 73±82 | 1.0 | 152±161 | 177±229 | 0.6 | 83±127 | 113±194 | 0.4 |
| **Total CD8^+^ T cells** | 310±206 | 317±191 | 0.9 | 677±503 | 671±500 | 0.9 | 382±450 | 393±416 | 0.9 |
| **Naïve CD8^+^ T cells (CCR7^+^ CD45RO^-^)** | 106±92 | 112±109 | 0.8 | 198±174 | 181±112 | 0.7 | 95±156 | 82±98 | 0.7 |
| **Central memory CD8^+^ T cells (CCR7^+^ CD45RO^+^)** | 33±27 | 28±18 | 0.3 | 79±79 | 69±58 | 0.5 | 48±74 | 44±56 | 0.8 |
| **Effector memory CD8^+^ T cells (CCR7^-^ CD45RO^+^)** | 123±126 | 121±131 | 0.9 | 204±282 | 226±435 | 0.8 | 84±233 | 114±340 | 0.7 |
| **Terminally differentiated CD8^+^ T cells (CCR7^-^ CD45RO^-^)** | 90±64 | 93±112 | 0.9 | 206±195 | 208±166 | 1.0 | 121±186 | 132±132 | 0.8 |
| **Total B cells (CD19^+^)** | 135±142 | 187±225 | 0.2 | 254±216 | 256±172 | 1.0 | 124±139 | 79±177 | 0.2 |
| **Naïve B cells (CD19^+^ CD27^-^)** | 109±98 | 147±179 | 0.2 | 212±159 | 219±173 | 0.8 | 107±144 | 82±186 | 0.2 |
| **Memory B cells (CD19^+^ CD27^+^)** | 42±92 | 40±55 | 0.9 | 69±94 | 61±43 | 0.7 | 28±66 | 23±47 | 0.9 |

**Table S4: PBMC subsets at baseline, at 6m and the change Δ (6m-0m) during the TNFi treatment according to clinical remission.** PBMC subsets measured by flow cytometry in absolute cell count (mean±SD). All the subset’s data followed a normal distribution. Unpaired t test was employed to analyse the differences between non-REM and REM patients. Significant statistical differences are noted in bold. P-value<0.05 was considered as statistically significant.
